# Neutrophil-to-lymphocyte ratio, platelet-to-lymphocyte ratio, and monocyte-to-lymphocyte ratio as prognostic indicators in *Pneumocystis jirovecii* pneumonia

**DOI:** 10.3389/fmed.2026.1763688

**Published:** 2026-02-09

**Authors:** Fei Yu, Yousheng Ye, Min Shao

**Affiliations:** 1 The First Affiliated Hospital of Anhui Medical University, Hefei, China; 2 The First Affiliated Hospital of University of Science and Technology of China, Hefei, China; 3 The First People’s Hospital of Hefei, Hefei, China

**Keywords:** neutrophil-to-lymphocyte ratio, platelet-to-lymphocyte ratio, *Pneumocystis jirovecii* pneumonia, prognosis, trajectory analysis

## Abstract

**Background:**

*Pneumocystis jirovecii* pneumonia (PJP) remains a life-threatening opportunistic infection with high mortality, particularly among non-HIV immunocompromised patients. Identifying accessible and reliable prognostic biomarkers is of major clinical importance.

**Objectives:**

To investigate the prognostic value of dynamic changes in the neutrophil-to-lymphocyte ratio (NLR), monocyte-to-lymphocyte ratio (MLR), and platelet-to-lymphocyte ratio (PLR) among patients with PJP.

**Methods:**

A retrospective study of 165 PJP patients was conducted at two tertiary hospitals. Post-diagnostic trajectories of NLR, MLR, and PLR were analyzed using group-based trajectory modeling (GBTM). Associations between these trajectories and 28-day survival were assessed by Cox proportional hazards regression and Kaplan–Meier survival analysis.

**Results:**

Three distinct NLR trajectories were identified: continuously decreasing (15%), stable (68%), and continuously increasing (17%). Patients with continuously decreasing NLR had significantly lower 28-day survival (*P* < 0.05). The log-transformed NLR (logNLR) trajectory was an independent prognostic factor, whereas logMLR and logPLR were not significantly associated with outcomes.

**Conclusion:**

The temporal trajectory of logNLR is strongly associated with 28-day survival in PJP. A persistently declining logNLR predicts poor prognosis, suggesting its utility in early risk stratification.

## Introduction

*Pneumocystis jirovecii* pneumonia (PJP) is a major opportunistic and life-threatening infection that primarily affects immunocompromised individuals or those undergoing long-term immunosuppressive or corticosteroid therapy (1–3). Although prophylactic treatment has reduced AIDS-related PJP, the incidence among non-HIV immunocompromised hosts continues to rise, accompanied by high mortality rates (4–6).

Inflammatory responses and immune function play crucial roles in PJP pathogenesis (7, 8). However, no specific biomarkers have been consistently associated with prognosis. Simple hematological ratios—such as neutrophil-to-lymphocyte ratio (NLR), monocyte-to-lymphocyte ratio (MLR), and platelet-to-lymphocyte ratio (PLR)—offer easily accessible, cost-effective, and repeatable markers reflecting the balance between innate and adaptive immunity. Nevertheless, few studies have examined their temporal evolution in PJP (9, 10).

To address this gap, we applied group-based trajectory modeling (GBTM) to analyze longitudinal logNLR, logMLR, and logPLR data from PJP patients, identifying trajectory groups and their association with 28-day survival (11, 12).

## Materials and methods

### Study design and population

A retrospective cohort of 165 patients diagnosed with *Pneumocystis jirovecii* pneumonia (PJP) between October 2020 and August 2025 was included. Diagnostic criteria: clinical symptoms (fever, cough, dyspnea), radiologic findings of pulmonary infiltrates, and positive *P. jirovecii* detection in bronchoalveolar lavage fluid via metagenomic next-generation sequencing (mNGS) (13). Exclusion: patients <18 years, HIV-positive status, and pregnancy or lactation.

Ethical approval was granted by both hospital committees. Written informed consent was obtained from all participants.

### Data collection

Clinical variables included demographics, comorbidities, hospital and ICU stays, cute physiology and chronic health evaluation II (APACHE II) scores and sequential Organ Failure Assessment (SOFA) scores, treatments, and laboratory indices (WBC, NEUT, LYM, MONO, PLT, CRP, PT, FIB, LDH, etc.). Dynamic values of neutrophil-to-lymphocyte ratio (NLR), monocyte-to-lymphocyte ratio (MLR), and platelet-to-lymphocyte ratio (PLR) were recorded up to 28 days post-diagnosis.

### Outcome measures

The primary endpoint was 28-day survival after PJP diagnosis.

### Statistical analysis

Continuous variables were assessed for normality using the Shapiro–Wilk test. Between-group comparisons used *t*-test or Mann–Whitney U-test. Categorical variables were compared by chi-square or Fisher’s exact test.

Group-based trajectory modeling was used to model trajectories for logNLR, logMLR, and logPLR in R (v4.3.2). The best-fitting model was selected based on AIC/BIC. Associations with mortality were analyzed via Cox regression and Kaplan–Meier analysis. Statistical significance was defined as two-sided *P* < 0.05.

## Results

### Baseline characteristics

Among 165 *Pneumocystis jirovecii* pneumonia (PJP) patients (101 male, 64 female), 128 survived and 37 died within 28 days. Non-survivors had longer Intensive Care Unit (ICU) stays, higher cute physiology and chronic health evaluation II (APACHE II) scores and sequential Organ Failure Assessment (SOFA) scores, and were more likely to receive vasoactive therapy. Laboratory markers including PT, D-dimer, CRP, LDH, and PCT were elevated in non-survivors, whereas lymphocyte and monocyte counts were lower (Table 1).

**TABLE 1:** Baseline characteristics of survivors and non-survivors with *Pneumocystis jirovecii* pneumonia (PJP).

Characteristics	Category	Group D	Group L	*P*
*n*	–	37	128	–
Male (%)	M	17 (45.9)	84 (65.6)	0.049
	W	20 (54.1)	44 (34.4)	–
Age (median [IQR])	–	67.00 [54.00, 73.00]	61.00 [53.00, 72.00]	0.297
LOS1 (median [IQR])	–	12.00 [8.00, 16.00]	12.77 [8.00, 24.00]	0.177
LOS2 (median [IQR])	–	6.00 [2.00, 10.00]	0.00 [0.00, 10.88]	0.013
Health (%)	0	5 (13.5)	30 (23.4)	0.541
	1	3 (8.1)	15 (11.7)	–
	2	9 (24.3)	21 (16.4)	–
	3	3 (8.1)	5 (3.9)	–
	4	4 (10.8)	16 (12.5)	–
	5	10 (27.0)	25 (19.5)	–
	6	3 (8.1)	16 (12.5)	–
APACHE II (median [IQR])	–	20.00 [15.00, 24.00]	8.00 [6.00, 17.00]	<0.001
SOFA (median [IQR])	–	4.00 [3.00, 6.00]	2.00 [0.00, 4.00]	<0.001
CT (%)	0	0 (0.0)	1 (0.8)	1
	1	37 (100.0)	127 (99.2)	–
Therapy1 (%)	0	16 (43.2)	71 (55.5)	0.261
	1	21 (56.8)	57 (44.5)	–
Therapy3 (%)	0	14 (37.8)	60 (46.9)	0.432
	1	23 (62.2)	68 (53.1)	–
Therapy4 (%)	0	14 (37.8)	86 (67.2)	0.002
	1	23 (62.2)	42 (32.8)	–
Therapy5 (%)	0	4 (10.8)	48 (37.5)	0.004
	1	33 (89.2)	80 (62.5)	–
Therapy6 (median [IQR])	–	6.00 [4.00, 10.00]	4.00 [0.00, 10.25]	0.105
Therapy7 (%)	0	5 (13.5)	71 (55.5)	<0.001
	1	32 (86.5)	57 (44.5)	–
Therapy8 (median [IQR])	–	0.00 [0.00, 14.00]	0.00 [0.00, 0.00]	0.002
Therapy9 (median [IQR])	–	93.00 [21.00, 193.00]	0.00 [0.00, 0.00]	<0.001
Therapy10 (%)	0	30 (81.1)	114 (89.1)	0.316
	1	7 (18.9)	14 (10.9)	–
Therapy11 (%)	0	33 (89.2)	122 (95.3)	0.325
	1	4 (10.8)	6 (4.7)	–
Therapy12 (%)	0	34 (91.9)	118 (92.2)	1
	1	3 (8.1)	10 (7.8)	–
Therapy13 (%)	0	32 (86.5)	101 (78.9)	0.549
	1	5 (13.5)	27 (21.1)	–
APTT (median [IQR])	–	28.35 [26.37, 38.83]	29.35 [26.64, 34.92]	0.911
FIB (median [IQR])	–	4.80 [3.47, 5.44]	4.36 [3.41, 5.62]	0.872
PT (median [IQR])	–	13.60 [11.95, 15.20]	12.70 [11.74, 13.50]	0.033
PCT (median [IQR])	–	0.89 [0.15, 1.78]	0.13 [0.05, 0.30]	0.002
LYMPH (median [IQR])	–	0.45 [0.31, 0.60]	0.74 [0.40, 1.25]	0.011
MONO (median [IQR])	–	0.23 [0.11, 0.33]	0.41 [0.22, 0.60]	0.001
NEUT (median [IQR])	–	6.82 [5.53, 9.93]	6.11 [3.99, 8.66]	0.161
WBC (median [IQR])	–	7.78 [6.32, 10.55]	7.22 [5.95, 10.20]	0.566
LAC (median [IQR])	–	2.00 [1.33, 3.32]	1.45 [1.12, 1.80]	0.084
PO_2_ (median [IQR])	–	64.55 [48.50, 89.95]	81.00 [70.00, 95.20]	0.124
FIO_2_ (median [IQR])	–	0.50 [0.50, 0.50]	0.21 [0.21, 0.23]	0.050
PO_2_/FIO_2_ (median [IQR])	–	153.60 [153.60, 153.60]	359.52 [284.66, 407.14]	0.181
ALB (median [IQR])	–	29.70 [25.57, 33.25]	32.80 [28.65, 36.00]	0.015
ALT (median [IQR])	–	21.00 [16.08, 30.25]	22.00 [14.00, 38.00]	0.742
DD (median [IQR])	–	4.40 [1.54, 9.81]	1.15 [0.50, 2.74]	0.001
TBIL (median [IQR])	–	12.25 [9.38, 17.11]	9.15 [6.60, 12.60]	0.05
CRP (median [IQR])	–	91.16 [79.23, 151.04]	29.26 [13.28, 61.19]	<0.001
LDH (median [IQR])	–	625.55 [497.75, 1092.88]	400.00 [221.50, 548.49]	<0.001
BUN/CREA (median [IQR])	–	32.07 [22.74, 56.85]	21.47 [15.45, 29.78]	0.003
CREA (median [IQR])	–	67.00 [47.35, 118.00]	68.00 [50.08, 101.30]	0.817
PLT (median [IQR])	–	146.00 [78.75, 206.50]	207.00 [141.00, 279.75]	0.013
ESR (median [IQR])	–	33.00 [26.00, 52.00]	46.00 [22.50, 67.50]	0.368
HCO_3_^–^ (median [IQR])	–	19.30 [17.05, 21.45]	25.32 [22.64, 27.66]	0.066
NLR (median [IQR])	–	13.29 [9.26, 23.77]	8.20 [3.38, 18.76]	0.012
MLR (median [IQR])	–	0.47 [0.27, 0.64]	0.51 [0.30, 0.83]	0.239
PLR (median [IQR])	–	294.01 [167.62, 437.08]	262.00 [168.60, 467.53]	0.966

LOS1, total hospitalization duration; LOS2, ICU stay duration; Health, baseline conditions (absent/graft/tears/hematologic diseases/pulmonary conditions/connected tissue diseases/diabetes: 0/1/2/3/4/5/6); CT, chest CT scan showing ground-glass opacity (present/absent: 1/0); Therapy1, antibiotic use within 1 week before admission (present/absent: 1/0); Therapy2, trimethoprim-sulfamethoxazole tablet dosage; Therapy3, carbenicin (present/absent: 1/0); Therapy4, antiviral drugs (present/absent: 1/0); Therapy5, glucocorticoid use (present/absent: 1/0); Therapy6, glucocorticoid treatment duration (days); Therapy7, vasopressors (present/absent: 1/0); Therapy8, non-invasive mechanical ventilation duration (hours); Therapy9, invasive mechanical ventilation duration (hours); Therapy10, CRRT (present/absent: 1/0); Therapy11, ECMO (present/absent: 1/0); Therapy12, fungal infection (present/absent: 1/0); Therapy13, cytomegalovirus (present/absent: 1/0); M, male; W, female; L, survived; D, died; WBC, white blood cell count; NEUT, neutrophil absolute count; LYM, lymphocyte absolute count; MONO, monocyte absolute count; PLT, platelet count; CRP, C-reactive protein (RRT); APTT, activated partial thromboplastin time; FIB, fibrinogen; PT, prothrombin time; D-D, D-dimer; PCT, procalcitonin; ESR, erythrocyte sedimentation rate; CREA, creatinine; BUN/CREA, blood urea nitrogen/creatinine; TBIL, total bilirubin; ALT, alanine aminotransferase; ALB, albumin; LDH, lactate dehydrogenase; HCO_3_^–^, carbon dioxide partial pressure; LAC, Lactic acid; PaO_2_, Partial oxygen pressure; FIO_2_, Fraction of oxygen administered; PO_2_/FIO_2_, Oxygenation index; NLR, Neutrophil/lymphocyte ratio; MLR, Monocyte/lymphocyte ratio; PLR, Platelet/lymphocyte ratio.

### LogNLR trajectories

**FIGURE 1:**
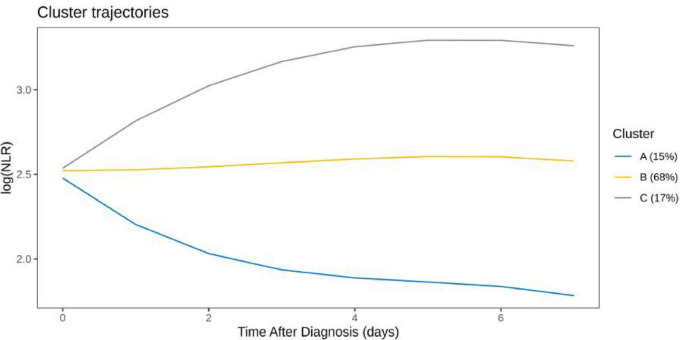
LogNLR trajectory comparison among clusters.

**FIGURE 2:**
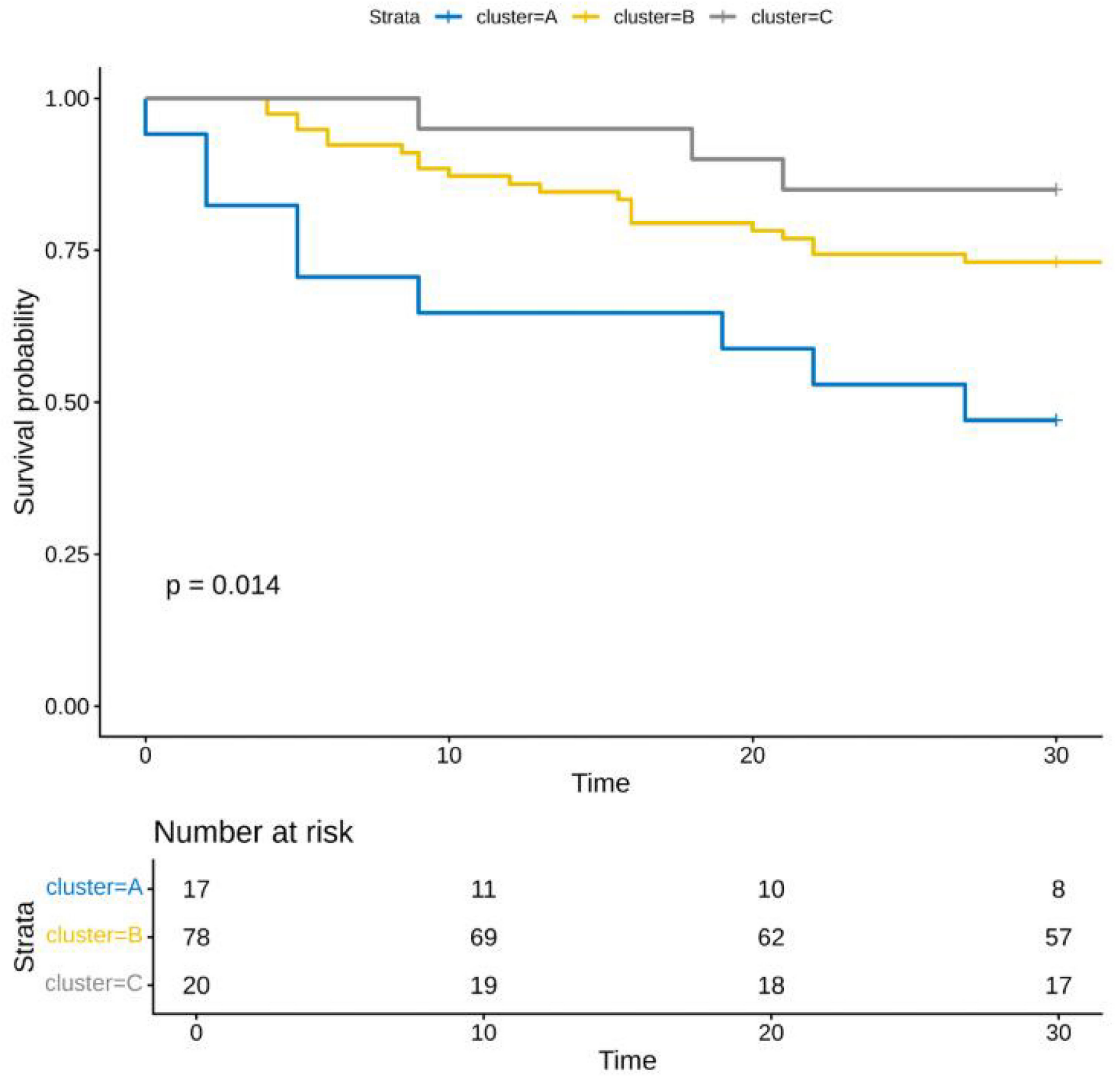
Kaplan–Meier survival curves by logNLR trajectory.

Three logNLR trajectories were identified: continuously decreasing (15%), stable (68%), and increasing (17%). Kaplan–Meier survival analysis revealed significantly lower survival in the decreasing group (*P* = 0.014) (Figures 1, 2).

### LogMLR and logPLR trajectories

**FIGURE 3:**
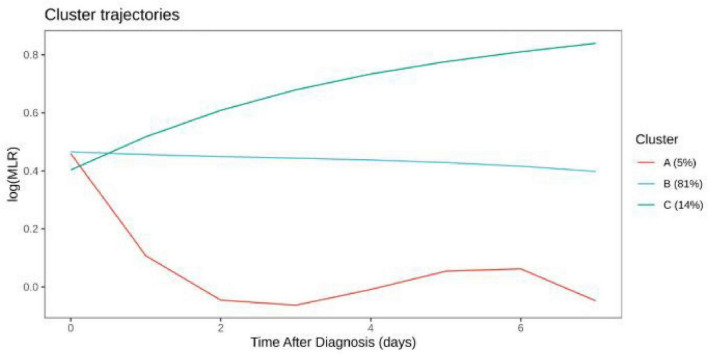
LogMLR trajectory comparison among clusters.

**FIGURE 4:**
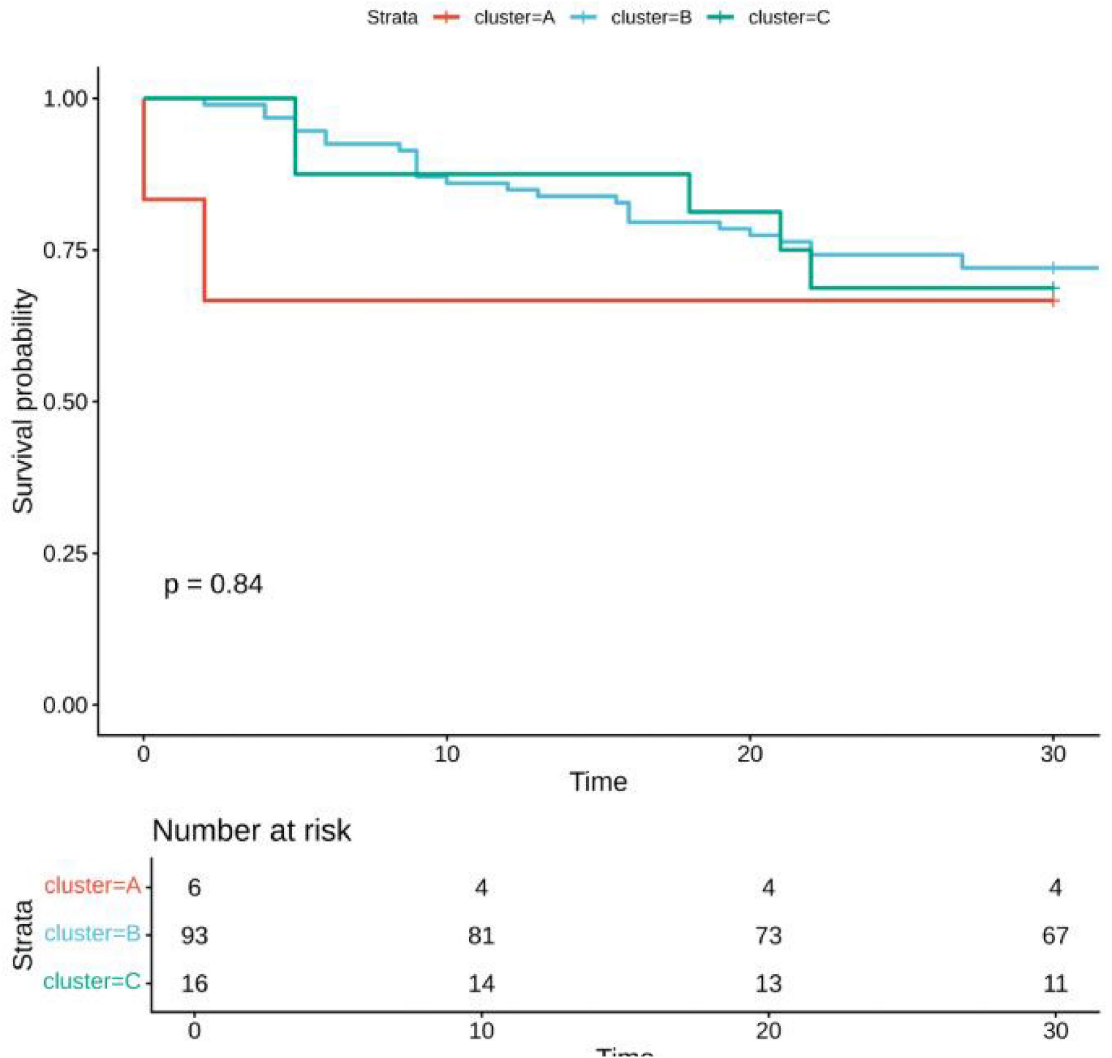
Survival analysis by logMLR trajectories.

**FIGURE 5:**
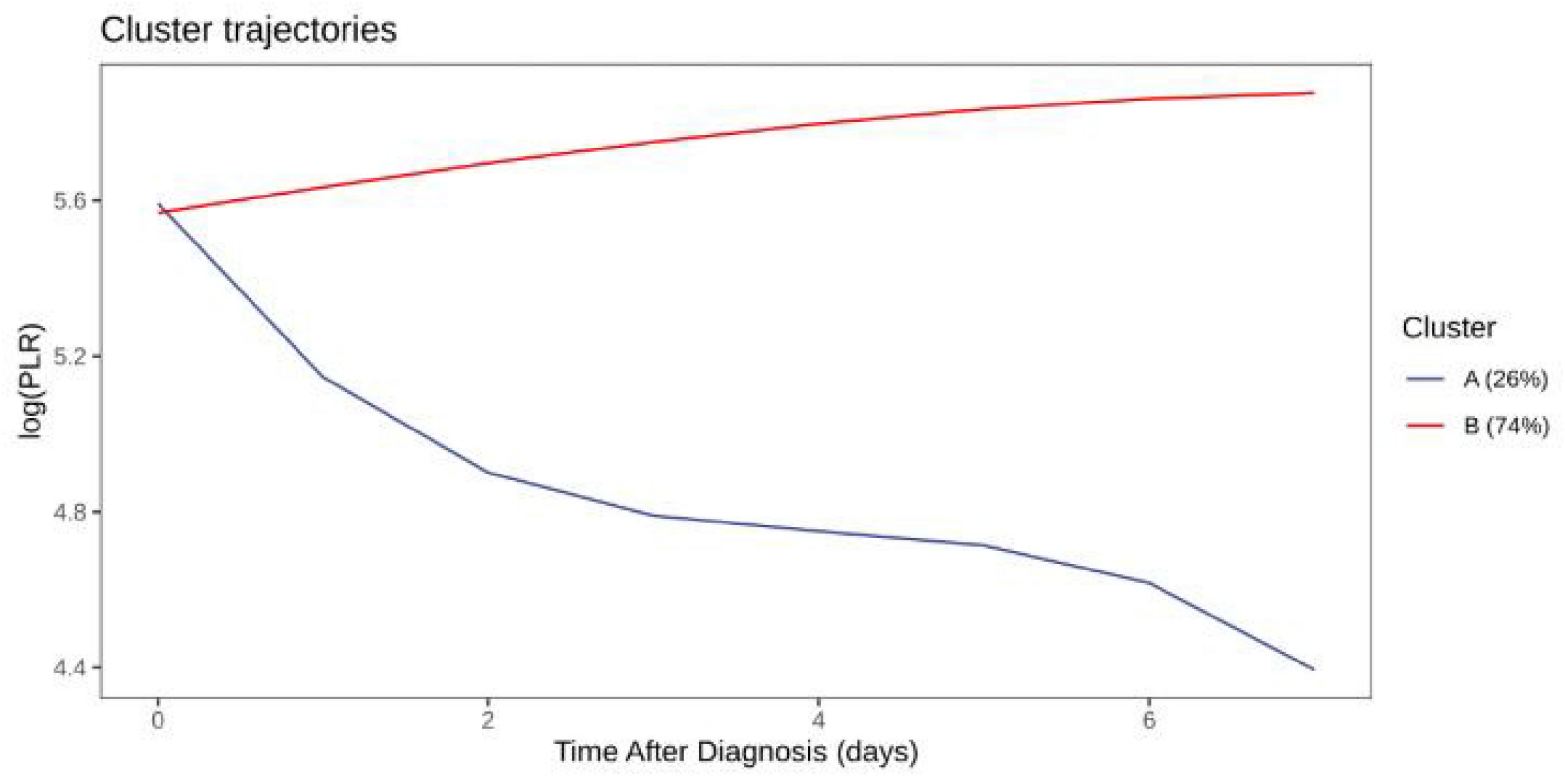
LogPLR trajectory comparison among clusters.

**FIGURE 6:**
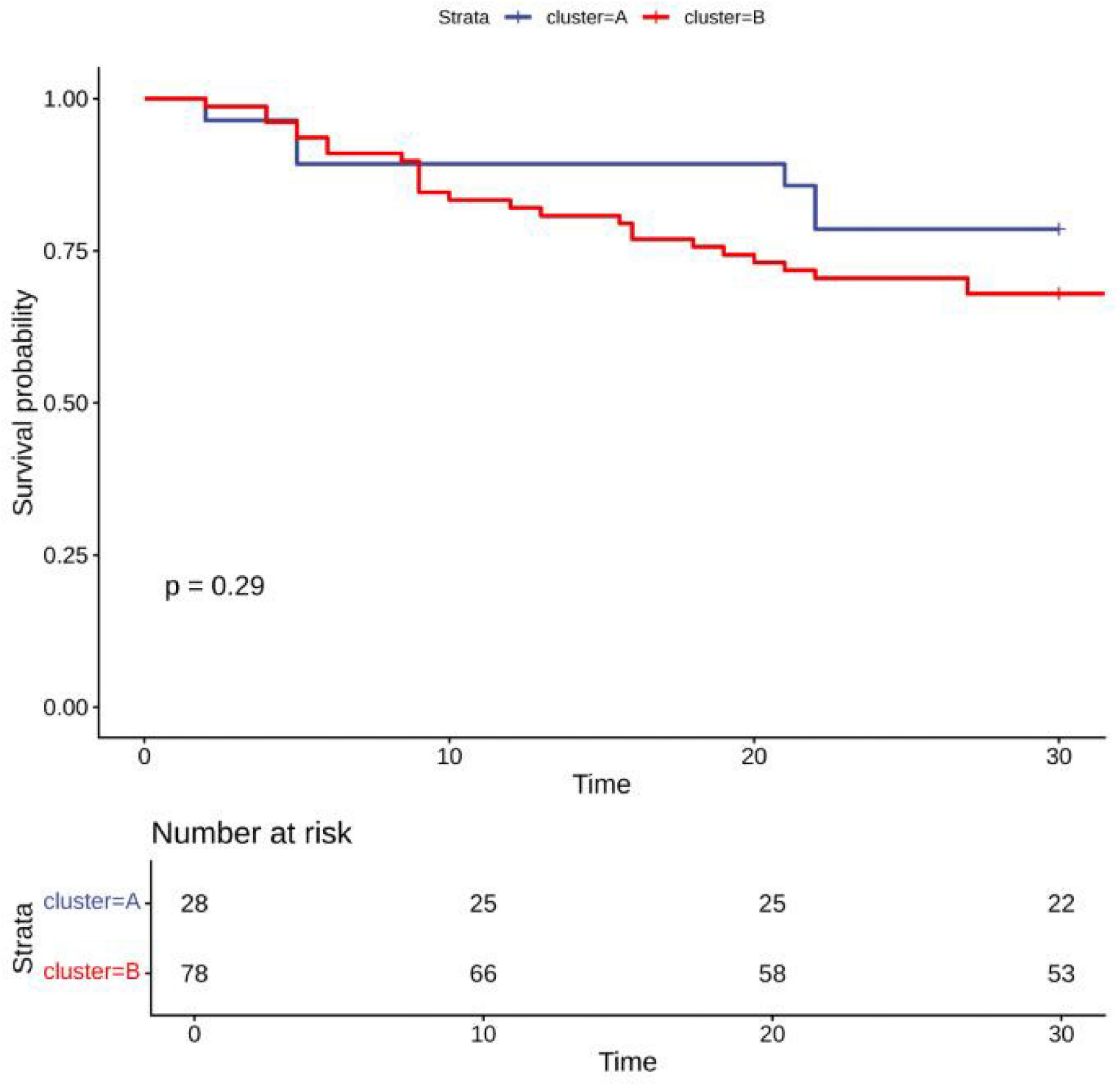
Survival analysis by logPLR trajectories.

Distinct logMLR and logPLR patterns were observed but showed no significant association with survival (logMLR *P* = 0.84; logPLR *P* = 0.29) (Figures 3–6).

### Clinical correlates

Patients in the decreasing logNLR group had higher SOFA scores and different corticosteroid usage patterns (*P* < 0.05) (Table 2).

**TABLE 2:** Baseline characteristics according to logNLR trajectory groups.

Characteristics	Category	Group A	Group B	Group C	*P*
*n*	–	18	79	20	–
Male (%)	M	10 (55.6)	54 (68.4)	9 (45.0)	0.127
	W	8 (44.4)	25 (31.6)	11 (55.0)	
Age (median [IQR])	–	55.50 [36.00, 67.00]	64.00 [53.50, 72.50]	59.00 [52.75, 68.25]	0.064
LOS1 (median [IQR])	–	18.00 [12.00, 37.25]	14.00 [9.00, 22.50]	12.77 [10.12, 20.75]	0.294
LOS2 (median [IQR])	–	4.82 [0.23, 17.70]	3.00 [0.00, 12.00]	6.50 [0.00, 12.16]	0.57
Health (%)	0	4 (22.2)	16 (20.3)	3 (15.0)	0.63
	1	4 (22.2)	8 (10.1)	1 (5.0)	–
	2	1 (5.6)	15 (19.0)	3 (15.0)	–
	3	2 (11.1)	2 (2.5)	2 (10.0)	–
	4	3 (16.7)	9 (11.4)	2 (10.0)	–
	5	3 (16.7)	19 (24.1)	6 (30.0)	–
	6	1 (5.6)	10 (12.7)	3 (15.0)	–
APACHE II (median [IQR])	–	16.00 [13.50, 23.00]	10.00 [6.00, 20.50]	15.50 [10.75, 21.00]	0.11
SOFA (median [IQR])	–	6.00 [4.00, 8.75]	2.00 [0.00, 5.00]	2.50 [1.50, 6.00]	0.002
CT (median [IQR])	–	1.00 [1.00, 1.00]	1.00 [1.00, 1.00]	1.00 [1.00, 1.00]	0.786
Therapy1 (%)	0	9 (50.0)	44 (55.7)	10 (50.0)	0.846
	1	9 (50.0)	35 (44.3)	10 (50.0)	–
Therapy3 (%)	0	4 (22.2)	33 (41.8)	6 (30.0)	0.237
	1	14 (77.8)	46 (58.2)	14 (70.0)	–
Therapy4 (%)	0	8 (44.4)	49 (62.0)	8 (40.0)	0.123
	1	10 (55.6)	30 (38.0)	12 (60.0)	–
Therapy5 (%)	0	3 (16.7)	26 (32.9)	1 (5.0)	0.024
	1	15 (83.3)	53 (67.1)	19 (95.0)	–
Therapy6 (median [IQR])	–	11.00 [4.50, 17.00]	6.00 [0.00, 12.00]	7.50 [3.75, 10.25]	0.189
Therapy7 (%)	0	4 (22.2)	33 (41.8)	7 (35.0)	0.293
	1	14 (77.8)	46 (58.2)	13 (65.0)	–
Therapy8 (median [IQR])	–	0.00 [0.00, 0.00]	0.00 [0.00, 2.50]	0.00 [0.00, 0.00]	0.177
Therapy9 (median [IQR])	–	89.50 [0.00, 245.25]	0.00 [0.00, 88.00]	0.00 [0.00, 99.00]	0.141
Therapy10 (%)	0	13 (72.2)	68 (86.1)	17 (85.0)	0.351
	1	5 (27.8)	11 (13.9)	3 (15.0)	–
Therapy11 (%)	0	16 (88.9)	71 (89.9)	20 (100.0)	0.321
	1	2 (11.1)	8 (10.1)	0 (0.0)	–
Therapy12 (%)	0	16 (88.9)	73 (92.4)	20 (100.0)	0.358
	1	2 (11.1)	6 (7.6)	0 (0.0)	–
Therapy13 (%)	0	14 (77.8)	63 (79.7)	19 (95.0)	0.233
	1	4 (22.2)	16 (20.3)	1 (5.0)	–
APTT (median [IQR])	–	33.90 [28.80, 38.70]	30.20 [26.28, 35.65]	27.15 [26.40, 28.30]	0.172
FIB (median [IQR])	–	4.28 [3.39, 5.48]	4.44 [3.47, 5.65]	5.02 [3.41, 5.56]	0.935
PT (median [IQR])	–	13.40 [12.20, 15.50]	12.85 [11.74, 13.83]	12.45 [12.00, 13.30]	0.226
PCT (median [IQR])	–	0.32 [0.12, 1.04]	0.18 [0.07, 1.02]	0.13 [0.10, 0.30]	0.681
LYMPH (median [IQR])	–	0.44 [0.19, 0.78]	0.60 [0.36, 1.11]	0.49 [0.29, 0.67]	0.217
MONO (median [IQR])	–	0.17 [0.12, 0.46]	0.34 [0.14, 0.68]	0.28 [0.16, 0.36]	0.29
NEUT (median [IQR])	–	7.27 [5.81, 9.35]	6.68 [4.39, 9.66]	6.97 [4.73, 8.18]	0.62
WBC (median [IQR])	–	7.81 [6.33, 10.57]	7.65 [6.23, 10.66]	7.83 [5.29, 9.35]	0.538
LAC (median [IQR])	–	1.52 [1.23, 2.07]	1.50 [1.35, 2.64]	1.80 [1.80, 2.20]	0.653
PO_2_ (median [IQR])	–	84.65 [71.05, 112.25]	73.83 [49.08, 90.70]	78.70 [63.98, 82.32]	0.501
ALB (median [IQR])	–	29.15 [24.60, 33.81]	32.35 [27.91, 34.88]	28.50 [24.22, 32.76]	0.157
ALT (median [IQR])	–	16.50 [11.03, 30.50]	22.90 [16.95, 30.55]	20.50 [15.00, 30.50]	0.525
DD (median [IQR])	–	3.38 [1.64, 9.45]	1.60 [0.68, 4.40]	1.38 [0.65, 3.41]	0.324
TBIL (median [IQR])	–	10.45 [5.99, 15.86]	10.20 [7.48, 13.50]	6.80 [6.20, 11.00]	0.201
CRP (median [IQR])	–	88.18 [60.84, 127.95]	45.80 [17.75, 95.72]	80.77 [30.53, 111.48]	0.252
LDH (median [IQR])	–	537.00 [402.50, 625.55]	464.25 [282.58, 773.55]	593.50 [457.30, 1092.75]	0.171
BUN/CREA (median [IQR])	–	21.95 [12.87, 44.31]	28.97 [20.66, 36.43]	24.44 [14.38, 42.74]	0.519
CREA (median [IQR])	–	68.50 [43.10, 144.25]	69.00 [51.45, 110.00]	55.00 [40.00, 109.58]	0.566
HCO_3_^–^ (median [IQR])	–	22.75 [20.31, 23.45]	24.90 [20.52, 28.82]	22.25 [19.90, 25.95]	0.569
PLT (median [IQR])	–	148.00 [50.25, 233.75]	204.00 [110.25, 280.00]	178.50 [138.75, 209.25]	0.229
ESR (median [IQR])	–	26.00 [18.00, 28.50]	42.50 [22.25, 67.75]	52.00 [46.00, 59.00]	0.066
FIO_2_ (median [IQR])	–	0.21 [0.21, 0.21]	0.21 [0.21, 0.29]	0.45 [0.45, 0.45]	0.285

LOS1, Total hospitalization duration; LOS2, ICU stay duration; Health, baseline conditions (absent/graft/tears/hematologic diseases/pulmonary conditions/connected tissue diseases/diabetes: 0/1/2/3/4/5/6); CT, chest CT scan showing ground-glass opacity (present/absent: 1/0); Therapy1, antibiotic use within 1 week before admission (present/absent: 1/0); Therapy2, trimethoprim-sulfamethoxazole tablet dosage; Therapy3, carbenicin (present/absent: 1/0); Therapy4, antiviral drugs (present/absent: 1/0); Therapy5, glucocorticoid use (present/absent: 1/0); Therapy6, glucocorticoid treatment duration (days); Therapy7, vasopressors (present/absent: 1/0); Therapy8, non-invasive mechanical ventilation duration (hours); Therapy9, invasive mechanical ventilation duration (hours); Therapy10, CRRT (present/absent: 1/0); Therapy11, ECMO (present/absent: 1/0); Therapy12, fungal infection (present/absent: 1/0); Therapy13, cytomegalovirus (present/absent: 1/0); M, male; W, female; L, survived; D, died; WBC, white blood cell count; NEUT, neutrophil absolute count; LYM, lymphocyte absolute count; MONO, monocyte absolute count; PLT, platelet count; CRP, C-reactive protein (RRT); APTT, activated partial thromboplastin time; FIB, fibrinogen; PT, prothrombin time; D-D, D-dimer; PCT, procalcitonin; ESR, erythrocyte sedimentation rate; CREA, creatinine; BUN/CREA, blood urea nitrogen/creatinine; TBIL, total bilirubin; ALT, alanine aminotransferase; ALB, albumin; LDH, lactate dehydrogenase; HCO_3_^–^, carbon dioxide partial pressure; LAC, lactic acid; PaO_2_, partial oxygen pressure; FIO_2_, fraction of oxygen administered.

## Discussion

*Pneumocystis jirovecii* pneumonia (PJP) remains a potentially fatal opportunistic infection, particularly among patients with non-acquired immunodeficiency syndrome (NADIS) (14–16). Compared with AIDS-related PJP, NADIS-associated PJP is characterized by a more acute onset, rapid clinical deterioration, and substantially higher mortality rates, which are often attributed to delayed diagnosis and insufficient targeted antimicrobial therapy (6, 17–19). Previous studies have demonstrated that PJP in non-HIV populations is frequently accompanied by excessive inflammatory responses within the alveolar microenvironment, leading to impaired gas exchange and subsequent multi-organ dysfunction (20–23). In this context, identifying reliable and easily accessible biomarkers for dynamic assessment of inflammatory and immune status is of critical importance. Although molecular diagnostic technologies have significantly improved the detection of *P. jirovecii* (24, 25), robust indicators for treatment monitoring and prognostic stratification are still lacking (19, 26–28).

Recently, complete blood count (CBC)-derived inflammatory indices, including neutrophil-to-lymphocyte ratio (NLR), monocyte-to-lymphocyte ratio (MLR), and platelet-to-lymphocyte ratio (PLR), have attracted increasing attention due to their cost-effectiveness and clinical feasibility (29–34). These indices reflect the balance between innate and adaptive immunity and have shown prognostic value in various inflammatory and infectious diseases. However, their role in PJP, especially from a longitudinal perspective, remains insufficiently explored (35).

Our study demonstrated that baseline NLR was significantly higher in non-survivors than in survivors, indicating that an excessive inflammatory burden at admission is associated with poor short-term outcomes. More importantly, through group-based trajectory modeling (GBTM) of log-transformed NLR values, we identified three distinct dynamic patterns: sustained decline, stable trajectory, and sustained increase. Unexpectedly, patients in the sustained decline trajectory group exhibited the worst survival outcomes, whereas those with sustained increase trajectories showed the most favorable prognosis. This paradoxical phenomenon highlights that not only the absolute value of NLR, but also its temporal evolution, is crucial for outcome prediction.

The biological explanation for this finding may be multifactorial. Neutrophils play a central role in the early innate immune response against pathogens, while lymphocytes, particularly CD4^+^ T cells, are indispensable for effective clearance of *P. jirovecii*. An elevated NLR at disease onset likely reflects a hyperinflammatory state combined with impaired adaptive immunity (36, 37). However, a rapid decline in NLR during disease progression may represent immune exhaustion, bone marrow suppression, extensive lymphocyte apoptosis, or immune reconstitution inflammatory syndrome (IRIS) (28). These processes may result in functional immune collapse, impaired pathogen clearance, and an increased risk of fatal outcomes.

In addition, patients in the sustained decline group exhibited significantly higher sequential Organ Failure Assessment (SOFA) scores, suggesting more severe organ dysfunction and systemic involvement. Differences in glucocorticoid use among trajectory groups further reflect the complex immunomodulatory effects of corticosteroids in PJP, which may exert both beneficial and detrimental effects on immune homeostasis, depending on timing, dose, and patient-specific immune status.

Of note, MLR and PLR, either at baseline or during dynamic follow-up, were not significantly associated with 28-day mortality in our cohort. This finding suggests that NLR, integrating signals from both neutrophils and lymphocytes, may serve as a more sensitive and disease-specific marker for immunoinflammatory imbalance in PJP.

To the best of our knowledge, this is the first study to systematically evaluate the prognostic value of dynamic NLR trajectories in patients with PJP. Our findings provide a novel framework for early risk stratification and individualized clinical management. Dynamic monitoring of NLR trajectories may help clinicians to identify high-risk patients at an earlier stage, optimize immunomodulatory strategies, and allocate critical care resources more precisely.

Several limitations should be acknowledged. First, this was a retrospective with a relatively limited sample size, which may introduce selection bias. Second, follow-up was restricted to 28 days, and long-term outcomes were not assessed. Third, laboratory testing intervals were not completely standardized, although this limitation was partially mitigated by trajectory modeling techniques. Finally, external validation in large-scale, prospective, multicenter cohorts is required before these findings can be translated into routine clinical practice.

## Conclusion

In patients with *Pneumocystis jirovecii* pneumonia, the dynamic trajectory of logNLR is strongly associated with short-term survival. A persistently decreasing logNLR indicates poor prognosis and warrants closer clinical monitoring and supportive interventions.

## Data Availability

The original contributions presented in this study are included in this article/supplementary material, further inquiries can be directed to the corresponding authors.
